# Laparoscopic extralevator abdominoperineal excision in distal rectal cancer patients: a retrospective comparative study

**DOI:** 10.1186/s12893-022-01865-9

**Published:** 2022-12-08

**Authors:** Zhiqiang Wang, Rui Liang, Dilimulati Yalikun, Jun Yang, Wenliang Li, Zhiyong Kou

**Affiliations:** 1 Department of Anorectal Surgery, The Second Hospital of Tianjin Medical University, Tianjin, 300211 China; 2 Department of Pathology, The Second Hospital of Tianjin Medical University, Tianjin, 300211 China; 3 Department of Oncology, The First Affiliated Hospital of Kunming Medical University, 295 Xichang Road, Kunming, 650032 Yunnan China

**Keywords:** Laparoscope, Extralevator abdominoperineal resection, Conventional abdominoperineal excision, Distal rectal cancer

## Abstract

**Background:**

At present, abdominoperineal excision with neoadjuvant chemoradiotherapy (nCRT) is one of the treatment modalities of distal rectal cancer. Our study analyzed the effects of laparoscopic extralevator abdominoperineal resection (ELAPE) compared with laparoscopic conventional abdominoperineal resection(cAPR) in the treatment of distal rectal cancer.

**Methods:**

Retrospective analysis was conducted on the clinicopathological data of 177 distal rectal cancer patients treated with a laparoscopic abdominoperineal resection between 2011 and 2018. The patients were divided into four groups as follows: ELAPE without nCRT (group A), cAPR without nCRT (group B), ELAPE with long-course nCRT (group C) and cAPR with long-course nCRT (group D).

**Results:**

Positive circumferential resection margin (CRM), local recurrence rate, 3-year disease-free survival (DFS) and 3-year overall survival (OS) did not differ between group A and group B. The rate of positive CRM in group C was lower than group D (4.4% vs. 11.9%, respectively), although the difference was not significant (P = 0.377). The 3-year local recurrence rate in group C was lower compared with group D (6.6% vs. 16.7%, respectively), although the difference was not significant (P = 0.135). Three-year DFS and 3-year OS were not different between groups C and D.

**Conclusions:**

This study showed that the effect of laparoscopic ELAPE in patients with low-risk rectal cancer is similar to laparoscopic cAPR, revealing that laparoscopic cAPR can be routinely selected for patients with low-risk rectal cancer. Furthermore, laparoscopic ELAPE has a tendency to reduce the rate of positive CRM and local recurrence in patients with high-risk rectal cancer. Laparoscopic ELAPE can be routinely considered for patients with high-risk rectal cancer.

## Background

Laparoscopic surgery can achieve less postoperative complications and faster postoperative recovery in patients with distal rectal cancer [1]. Moreover, laparoscopic surgery can be performed safely in the elderly population, and reduce their pain and convalescence [2]. Therefore, laparoscopic technology has been routinely used in abdominoperineal excision (APE) in patients with distal rectal cancer [3–6].

During conventional abdominoperineal resection (cAPR) the mesorectum is mobilized along the surface of mesorectal fascia from the levator muscles down to the top of anorectal ring. Incision of the gap between mesorectum and levator muscles close to levator hiatus increases the risk of positive circumferential resection margin (CRM). To decrease the risk of positive CRM the mesorectum is not mobilized from the levator muscles and the gap between mesorectum and levator muscles is not incised in extralevator abdominoperineal resection (ELAPE). Compared with cAPR, ELAPE was considered to be able to reduce risk for intraoperative perforation (IOP), and positive CRM, which may induce local recurrence of tumor [7, 8]. At present, ELAPE has been suggested to be selectively performed in patients with advance stage distal rectal cancer [9].

Similarly, the incision of the gap between mesorectum and levator muscles much closer to rectal muscle tube in cAPR might increase the risk of positive CRM in early stage distal rectal cancer, too. Therefore, we speculate that ELAPE can also decrease the risk of positive CRM and influence the survival of the patient with early stage distal rectal cancer. In this study the data of patients undergoing cAPR and ELAPE were analyzed to evaluate the efficacy of ELAPE in patients with early stage distal rectal cancer.

In order to reduce tumor mass and local recurrence rate, neoadjuvant chemoradiotherapy (nCRT) is routinely applied to the treatment of patients with advance stage distal rectal cancer [10–13]. For patients with early stage distal rectal cancer, which includes T_1-3b_N_0_ determined by magnetic resonance imaging (MRI), surgery can be delivered directly [14]. For patients with advance stage distal rectal cancer, which includes T_3c-3d_N_0_, T_1-3_N_1-2_ and T_4b_, nCRT should be delivered before surgery [15].

In previous studies, patients with distal rectal cancer who received laparoscopic cAPR directly and those who underwent nCRT and then laparoscopic cAPR were seldom distinguished [16, 17]. To investigate the effect of laparoscopic ELAPE with or without nCRT, we divided patients into four groups according to whether they received long-course nCRT. The effects of laparoscopic ELAPE were analyzed using a stratified method compared with laparoscopic cAPR.

## Methods

### Patients

A total of 177 patients with distal rectal cancer treated with a curative laparoscopic ELAPE or laparoscopic cAPR at the Second Hospital of Tianjin Medical University (Tianjin, China) and the First Affiliated Hospital of Kunming Medical University (Kunming, China) were retrospectively reviewed between June 2011 and June 2018.

All patients were diagnosed with colonoscopy and pathological biopsy proved distal rectal adenocarcinoma. Clinical staging was evaluated by enhanced computed tomography (CT) and no distal metastasis was confirmed. T and N stages of rectal lesions were evaluated by magnetic resonance imaging (MRI) before surgery. Results of MRI evaluation in all patients showed that a safe distal margin could not be achieved using a sphincter sparing technique or tumor invasion in the anal sphincter.

For patients with low-risk rectal cancer, which included T_1-3b_N_0_ determined by MRI imaging, laparoscopic ELAPE (group A) or cAPR (group B) were delivered directly. Postoperative chemotherapy (mfolfox6 or Xelox) was recommended according to the final pathology report.

For patients with high-risk rectal cancer, which include T_3c-3b_N_0_, T_1-3_N_1-2_ and T_4b_ with MRF invasion, long-course nCRT was delivered before surgery. A total of 50.4–54.0 Gy of radiation and 5-FU-based chemotherapy (capecitabine) were given to patients. Laparoscopic ELAPE (group C) or cAPR (group D) were performed 8–10 weeks following nCRT. During the interval between nCRT and surgery, the patient received Xelox (2 times) or mfolfox6 (3 times) chemotherapy. Postoperative chemotherapy (mfolfox6 or Xelox) was recommended for 2–3 months.

### Operation procedure

The procedure of laparoscopic ELAPE and cAPR was conducted according to the principle of total mesorectal excision.

**Fig. 1:**
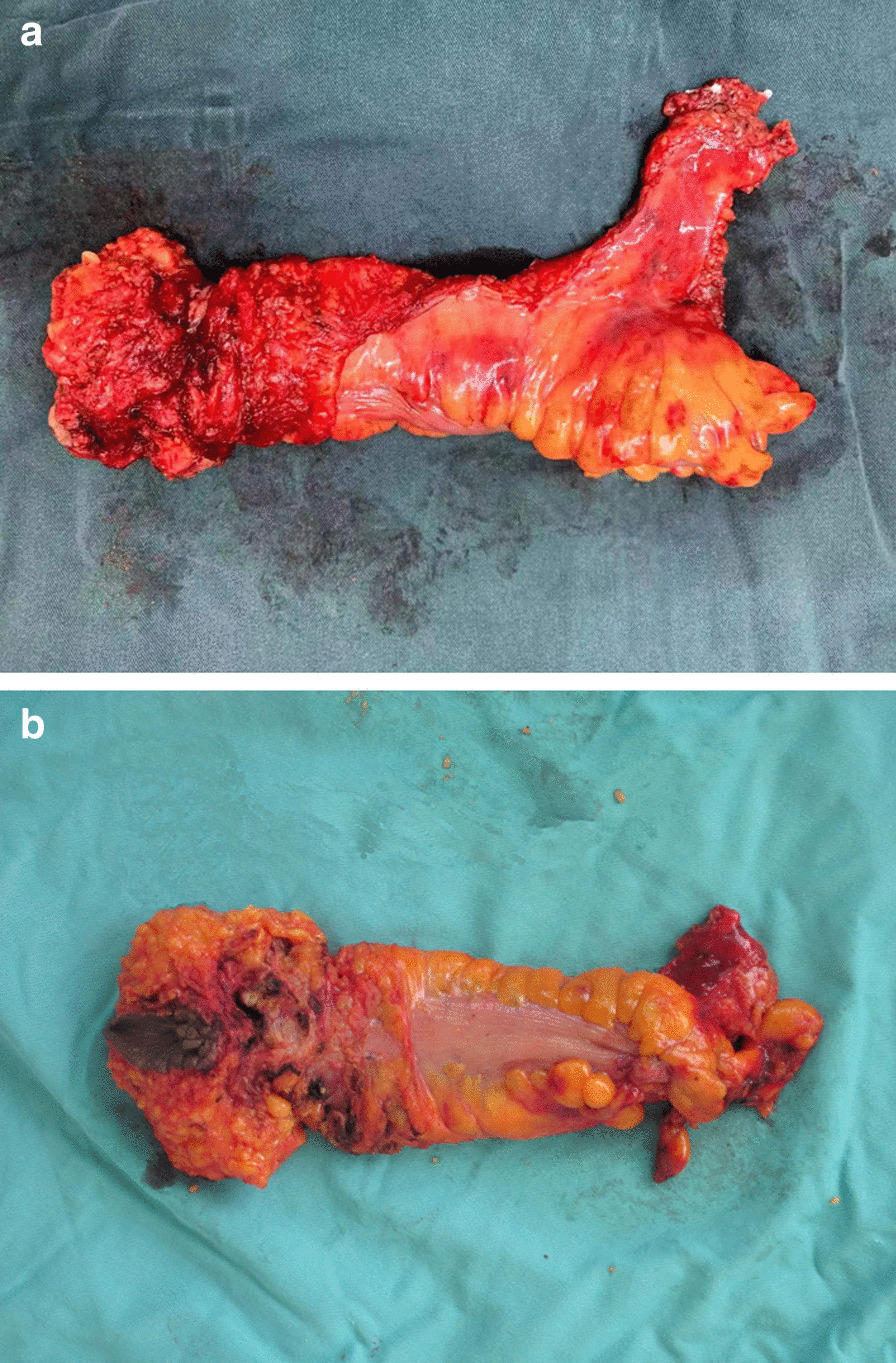
**a** Specimen of ELAPE The proper fascia of rectum is intact and the anorectal junction is completely covered by the levator ani muscle. **b** Specimen of cAPR The anorectal junction is not covered by the levator ani muscle

In laparoscopic ELAPE the mesorectum is not mobilized from the levator ani muscles, and the potential space between the mesorectum and levator ani muscle was not incised (Fig. 1). Therefore, after mobilizing the mesorectum along the surface of mesorectal fascia down to the beginning of the levator ani muscles the mobilization is stopped at the upper border of the coccyx posteriorly, just at the starting point of the levator ani muscle laterally and anteriorly. The rest of procedure was conducted as described in the previous literature [16, 18].

In laparoscopic cAPR the mesorectum is mobilized from the levator ani muscles, the potential space between the mesorectum and levator ani muscle was incised. Therefore, the mesorectum is mobilized along the surface of mesorectal fascia from the levator muscles down to the top of anorectal ring. The rest of procedure was conducted as described in the previous literature [16, 18].

### Outcome measures

Patient demographics, preoperative MRI-stage, neoadjuvant treatment, intraoperative perforation, pathologic stage, positive CRM, postoperative complications, local recurrence, 3-year disease-free survival (DFS) and 3-year overall survival (OS) were collected and analyzed.

### Follow-up

All patients were followed up in the two hospitals for an average of 36 months postoperatively. CEA, CT, MRI and Colonoscopy was conducted as described in the literature [19].

### Statistics

SPSS software (version 23.0; IBM Corp., Armonk, NY, USA) was applied for statistical analysis. Categorical data were analyzed using the Chi-square test. The Kaplan Meier method were used to calculate cumulative rates for local recurrence and survival. P < 0.05 was considered to be statistically significant.

## Results

The study included 177 patients; 90 patients did not receive nCRT, of which 49 cases underwent laparoscopic ELAPE (group A) and 41 cases underwent laparoscopic cAPR (group B). Characteristics of patients were listed in Tables 1 and 2. Gender, age and preoperative MRI-stage were not significantly different between group A and group B (Table 2). A total of 87 patients received nCRT before surgery, of which 45 cases underwent laparoscopic ELAPE (group C) and 42 cases underwent laparoscopic cAPR (group D). Gender, age, preoperative MRI-stage and neoadjuvant treatment were not significantly different between group C and group D (Table 2).

**Table 1:** Characteristics of patients after ELAPE and cAPR regardless of nCRT

	ELAPE (94)	cAPR (83)	P values
Age (years)	59.61 ± 11.93	58.33 ± 10.86	0.585
Gender			
Male	52	46	0.989
Female	42	37	
MRI-stage			
I	20	16	0.742
II	48	39	0.588
III	26	28	0.381
Neoadjuvant treatment	45	42	0.717

**Table 2:** Characteristics of patients

	Group A (49)	Group B (41)	P values	Group C (45)	Group D (42)	P values
Age (years)	60.22 ± 12.84	62.22 ± 12.07	0.453	59.28 ± 11.02	54.50 ± 10.21	0.537
Gender			0.921			0.894
Male	28	23		24	23	
Female	21	18		21	19	
MRI-stage			0.863			0.393
I	20	16		0	0	
II	29	25		19	14	
III	0	0		26	28	
Neoadjuvant Treatment	0	0	1.0	45	42	1.0

### Perioperative data

Perioperative data of the patients including intraoperative perforation, pathologic stage, positive CRM and postoperative complications collected from patient chart notes were listed in Tables 3 and 4.

**Table 3:** Perioperative data of patients after ELAPE and cAPR regardless of nCRT

	ELAPE (94)	cAPR (83)	P values
Intraoperative perforation	1	4	0.294
Pathologic stage			
pT0	0	0	1.0
pT1	12	6	0.224
pT2	37	35	0.704
pT3	0	0	1.0
pT4	0	0	
pN0	0	0	
pN1	0	0	
pN2	0	0	
ypT0	3	4	0.867
ypT1	7	6	0.956
ypT2	9	8	0.988
ypT3	21	17	0.764
ypT4	5	7	0.411
ypN0	21	19	0.930
ypN1	15	16	0.562
ypN2	9	7	0.792
Positive CRM	2	5	0.347
Postoperative complications			
Urinary retention	6	6	0.823
Intestinal obstruction	3	5	0.587
Perineal wound dehiscence	8	7	0.985

**Table 4:** Comparison of perioperative data from patients with distal rectal cancer

	Group A (49)	Group B (41)	P values		Group C (45)	Group D (42)	P values
Intraoperative perforation	0	0	1.0		1	4	0.317
Pathologic stage							
pT0				ypT0	3	4	0.924
pT1	12	6	0.244	ypT1	7	6	0.868
pT2	37	35	0.244	ypT2	9	8	0.911
pT3				ypT3	21	17	0.561
pT4				ypT4	5	7	0.453
pN0				ypN0	21	19	0.894
pN1	0	0	1.0	ypN1	15	16	0.643
pN2	0	0	0.244	ypN2	9	7	0.688
Positive CRM	0	0	1.0		2	5	0.377
Postoperative complications							
Urinary retention	3	1	0.742		3	5	0.636
Intestinal obstruction	1	2	0.875		2	3	0.937
Perineal wound dehiscence	3	4	0.806		5	3	0.788

No intraoperative perforation or positive CRM were observed in both group A and group B (P = 1.0). Pathologic stage was not significantly different between group A and group B. No unplanned reoperations or deaths were seen in group A or group B. The rate of intraoperative perforation in group C was lower compared with group D (1/45 vs. 4/42, respectively), although the difference was not significant (P = 0.317). The rate of positive CRM in group C was lower compared with group D (2/45 vs. 5/42, respectively), although the difference was not significant (P = 0.377). Pathological stage was not significantly different between group C and group D. No unplanned reoperations and deaths were seen in group C or group D (Table 4).

Postoperative intestinal obstruction, urinary retention, and perineal wound dehiscence were all comparable between group A and group B (P > 0.05). In addition, postoperative intestinal obstruction, urinary retention, and perineal wound dehiscence were all comparable between group C and group D (P > 0.05).

### Local recurrence

**Fig. 2:**
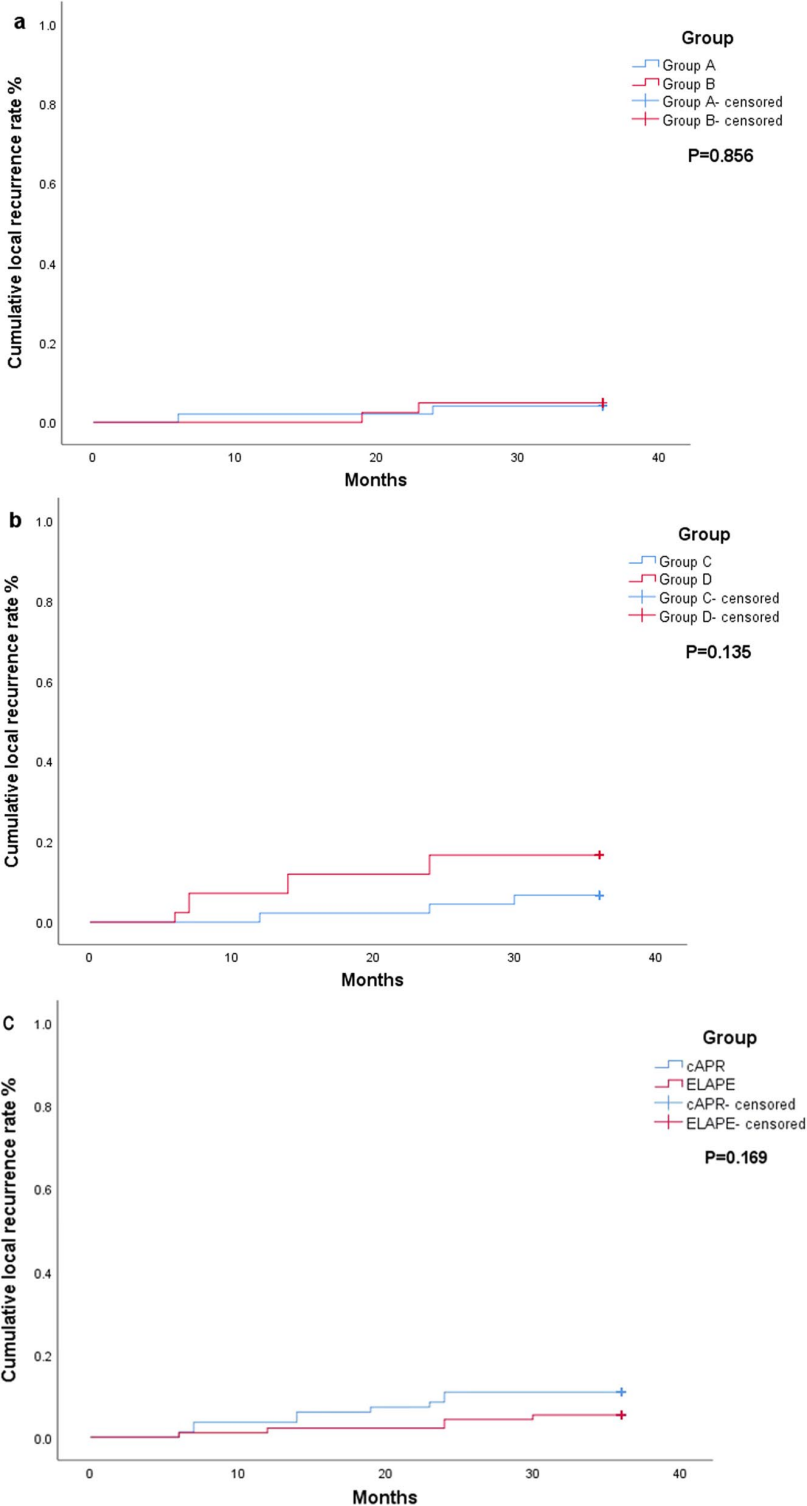
**a** Cumulative local recurrence rate of patients without nCRT. **b** Cumulative local recurrence rate of patients with nCRT. **c** Comparative analysis of cumulative local recurrence rate between ELAPE and cAPR patients regardless of nCRT. Dotted lines represent Kapan–Meier analysis. **a** P = 0.856, **b** P = 0.135, **c** P = 0.169

A total of 47 patients were alive without local recurrence at last follow-up in group A. Two patients developed local recurrence, and the 3-year local recurrence rate was 4.1% (2/49). Thirty-nine patients were alive without local recurrence at last follow-up in group B. Two patients developed local recurrence, and the 3-year local recurrence rate was 4.8% (2/41). The local recurrence rate was not significantly different between group A and group B (P = 0.856). The cumulative incidence curves are shown in Fig. 2.

A total of 41 patients were alive without local recurrence at last follow-up in group C. Three patients developed local recurrence, the local recurrence rate was 6.6% (3/45). Thirty-four patients were alive without local recurrence at last follow-up in group D. Seven patients developed local recurrence, and the local recurrence rate was 16.7% (7/42). The local recurrence rate was not significantly different between group C and group D (P = 0.135). The cumulative incidence curves are shown in Fig. 2.

### DFS

**Fig. 3:**
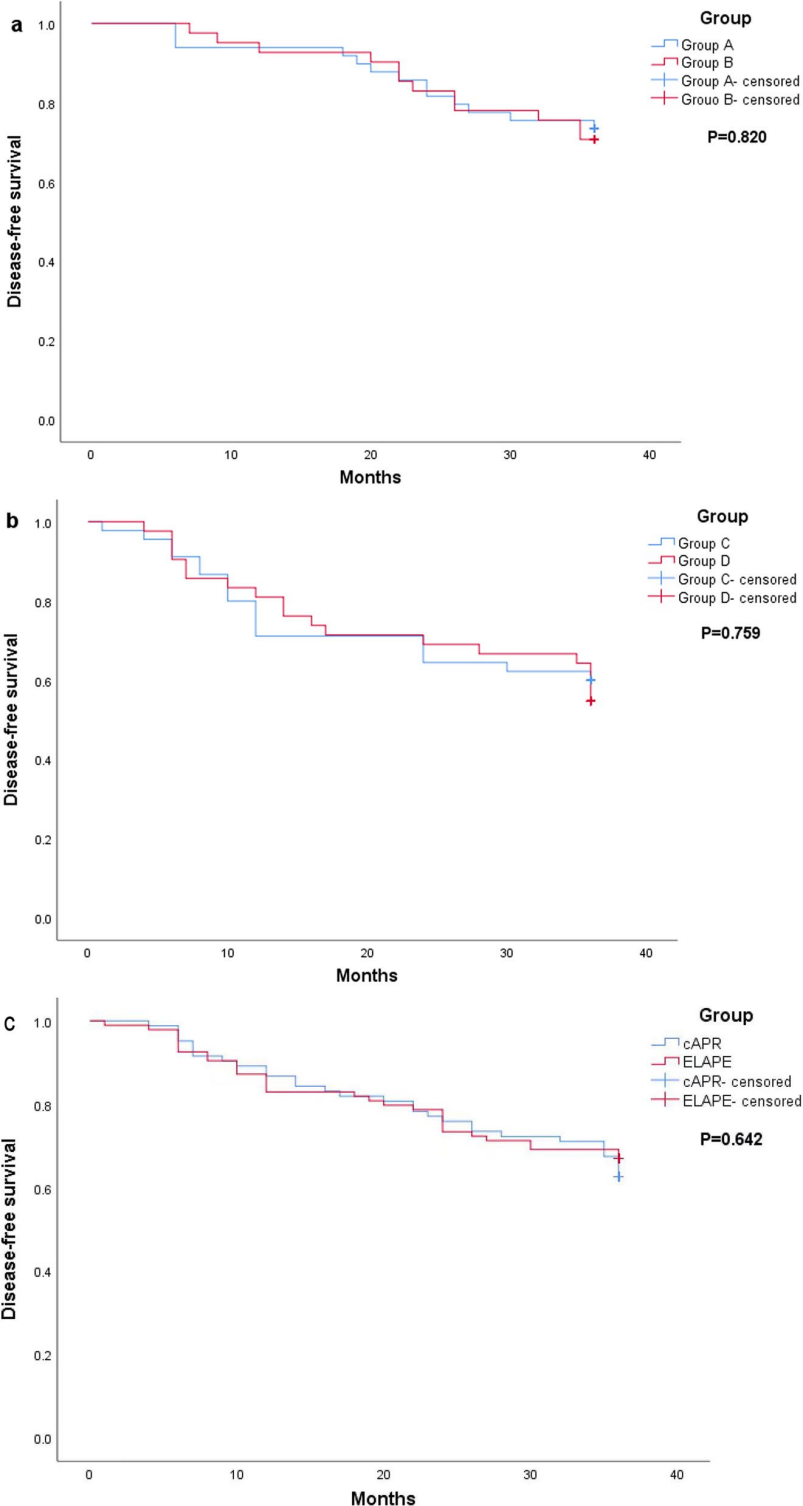
**a** 3-year DFS of patients without nCRT. **b** 3-year DFS of patients with nCRT. **c** Comparative analysis of 3-year DFS between ELAPE and cAPR patients regardless of nCRT. Dotted lines represent Kapan–Meier analysis. **a** P = 0.820, **b** P = 0.759, **c** = 0.642

In group A, two patients had local recurrence, four had liver metastasis, five had lung metastasis and two had liver and lung metastasis. The 3-year DFS rate was 73.5% (36/49) in group A. In group B, two patients had local recurrence, three had liver metastasis, six had lung metastasis and one had bone metastasis. The 3-year DFS rate was 70.7% (29/41) in group B. No difference in 3-year DFS was observed between groups A and B (P = 0.820) (Fig. 3).

In group C, three patients had local recurrence, five had liver metastasis, six had lung metastasis, three had liver and lung metastasis and one had bone metastasis. The 3-year DFS rate was 59.2% (27/45) in group C. In group D, five patients had local recurrence, two had local recurrence and liver metastasis, four had liver metastasis, five had lung metastasis, one had liver and lung metastasis and two had bone metastasis. The 3-year DFS rate was 54.8% (23/42) in group D. No difference in 3-year DFS was observed between groups C and D (P = 0.759) (Fig. 3).

### OS

**Fig. 4:**
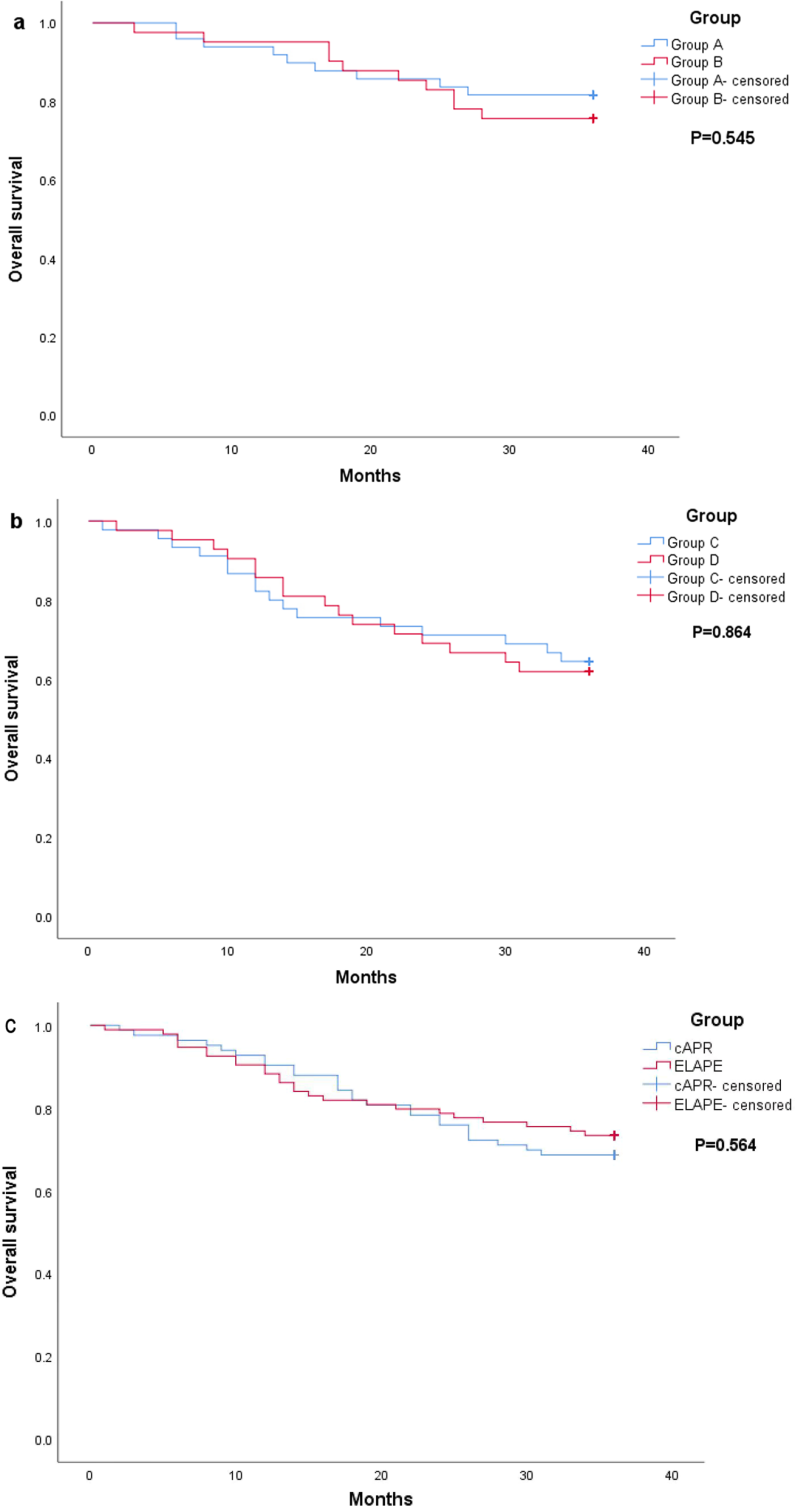
**a** 3-year OS of patients without nCRT. **b** 3-year OS of patients with nCRT. **c** Comparative analysis of 3-year OS between ELAPE and cAPR patients regardless of nCRT. Dotted lines represent Kapan-Meier analysis. **a** P = 0.545, **b** P = 0.864, **c** = 0.564

At 36 months follow-up, the median OS was not reached in either groups A or B. The 3-year OS survival rates were 81.6% (40/49) in group A and 75.6% (31/41) in group B, and the 3-year OS was not significantly different (P = 0.545) (Fig. 4).

In addition, the median OS was not reached in either groups C or D. The 3-year OS survival rates were 64.4% (29/45) in group C and 61.9% (26/42) in group D, and the 3-year OS was not significantly different (P = 0.864) (Fig. 4).

## Discussion

The extralevator abdominoperineal resection (ELAPE) lays emphasis on precise anatomy and complies with the radical resection of tumors principle. ELAPE has provided higher quality LN harvests and reduced the rate of intraoperative perforation, positive CRM and local recurrence compared with the conventional abdominoperineal resection(cAPR) procedure [20–25].

During the abdominal laparoscopic ELAPE procedure, the anterior space of the rectum does not require an incision to the hiatus in the levator ani muscle. However, the anterior lobe of the Denonvilliers fascia should be incised, otherwise the neurovascular bundles (NVB) on both sides of the seminal vesicle gland may be pulled and deformed during perineum operation. The NVB can be easily injured, which can lead to postoperative autonomic nerve dysfunction. In this study, the NVB in all patients within each of the four groups were effectively protected and no severe urinary or sexual function-related adverse events were observed.

MRI is used to determine which surgery, with or without nCRT, is routinely suggested for patients with rectal cancer [26–28]. In the present study following MRI, the patients in group A and group B underwent surgery without nCRT, and no positive CRM or intraoperative perforations were observed in either group. These results indicated that both laparoscopic APR and laparoscopic ELAPE can achieve satisfactory CRM and intraoperative perforation results in patients with low-risk rectal cancer.

The rate of intraoperative perforations and positive CRM in group C was lower than group D, although the difference was not significant. These results indicated that laparoscopic ELAPE has a tendency to reduce the rate of intraoperative perforation and positive CRM in patients with high-risk rectal cancer.

Laparoscopic ELAPE did not reduce local recurrence rate in patients with low-risk rectal cancer compared with laparoscopic cAPR, which indicated that a decreased local recurrence rate can be obtained by choosing either laparoscopic cAPR or laparoscopic ELAPE in patients with low-risk rectal cancer. The rate of local recurrence in group C was significantly lower than group D, although the difference was not significant. These findings indicated that laparoscopic ELAPE has a tendency to reduce local recurrence rate of patients with high-risk rectal cancer.

Laparoscopic ELAPE did not improve 3-year DFS or 3-year OS in patients with low-risk rectal cancer compared with laparoscopic cAPR. These results indicated different laparoscopic surgery did not influence the outcome of DFS or OS in patients with low-risk rectal cancer.

Although the results of our study reveal that laparoscopic ELAPE has a tendency to reduce the rate of intraoperative perforation, positive CRM and local recurrence rate of patients with high-risk rectal cancer, laparoscopic ELAPE did not improve 3-year DFS or 3-year OS in patients with high-risk rectal cancer compared with laparoscopic cAPR. This finding may be related to the increased proportion of patients with distant metastasis. The treatment course before surgery is approximately 3 months, thus with the extension of the course of treatment, the risk of distant metastasis is increased. Therefore, the effect of ELAPE for improving DFS and OS of patients with high-risk rectal cancer was influenced by nCRT.

With the rapid development of laparoscopic technology, the number of distal rectal cancer patients who underwent abdominoperineal resection shows a downward trend.

In order to obtain a sufficient number of cases, we collected the date of the patients from two medical centers. Therefore, all the operations were not performed by the same surgeon, which may have an impact on our research results.

In conclusion, the effect of laparoscopic ELAPE in patients with low-risk rectal cancer is similar to laparoscopic cAPR, revealing that laparoscopic cAPR can be routinely selected for patients with low-risk rectal cancer. Furthermore, laparoscopic ELAPE has a tendency to reduce the rate of positive CRM and local recurrence in patients with high-risk rectal cancer. However, laparoscopic ELAPE did not achieve a higher 3-year DFS or 3-year OS in patients with high-risk rectal cancer, which was related to an increased risk of distant metastasis caused by long-term nCRT treatment before surgery. Our results revealed that laparoscopic ELAPE can be routinely considered for patients with high-risk rectal cancer.

## Data Availability

The primary data analyzed during the current study are available from the corresponding author on reasonable request.

## Abbreviations

APE: Abdominoperineal excision
cAPR: Conventional abdominoperineal resection
CRM: Circumferential resection margin
ELAPE: Extralevator abdominoperineal resection
nCRT: Neoadjuvant chemoradiotherapy
MRI: Magnetic resonance imaging
MRF: Mesorectal fascia
CT: Computed tomography
MRI: Magnetic resonance imaging
